# Low Serum Dehydroepiandrosterone and Dehydroepiandrosterone Sulfate Are Associated With Coronary Heart Disease in Men With Type 2 Diabetes Mellitus

**DOI:** 10.3389/fendo.2022.890029

**Published:** 2022-06-27

**Authors:** Xinxin Zhang, Jinfeng Xiao, Tong Liu, Qing He, Jingqiu Cui, Shaofang Tang, Xin Li, Ming Liu

**Affiliations:** Department of Endocrinology and Metabolism, Tianjin Medical University General Hospital, Tianjin, China

**Keywords:** dehydroepiandrosterone, dehydroepiandrosterone sulfate, coronary heart disease, stroke, type 2 diabetes mellitus

## Abstract

**Aims:**

Sex hormones play an important role in the pathogenesis of cardiovascular disease (CVD). This cross-sectional study aimed to explore the associations of dehydroepiandrosterone (DHEA) and dehydroepiandrosterone sulfate (DHEAS) with coronary heart disease (CHD) and stroke in middle-aged and elderly patients with type 2 diabetes mellitus (T2DM).

**Materials and Methods:**

A total of 995 patients with T2DM were included in the study analysis. Serum levels of DHEA and DHEAS were quantified using liquid chromatography–tandem mass spectrometry. Binary logistic regression analyses were performed to assess the associations of DHEA and DHEAS with CHD and stroke. Receiver operating characteristic (ROC) curve analysis was performed to determine the optimal DHEA and DHEAS cutoff values for the detection of CHD in men with T2DM.

**Results:**

In men with T2DM, after adjustment for potential confounders in model 3, the risk of CHD decreased with an increasing serum DHEA level [odds ratio (OR) = 0.38, quartile 4 *vs*. quartile 1; 95% confidence interval (CI) = 0.16–0.90; *p* = 0.037 for trend). Consistently, when considered as a continuous variable, this association remained significant in the fully adjusted model (OR = 0.59, 95% CI = 0.40–0.87, *p* < 0.05). When taken as a continuous variable in model 3, serum DHEAS level was also inversely related to the risk of CHD among men (OR = 0.56, 95% CI = 0.38–0.82, *p* < 0.05). Similarly, this relationship remained statistically significant when DHEAS was categorized into quartiles (OR = 0.27, quartile 4 *vs*. quartile 1; 95% CI = 0.11–0.67; *p* = 0.018 for trend). ROC curve analyses revealed that the optimal cutoff values to detect CHD in men with T2DM were 6.43 nmol/L for DHEA and 3.54 μmol/L for DHEAS. In contrast, no significant associations were found between DHEA and DHEAS on the one hand and stroke on the other in men and women with T2DM (all *p* > 0.05).

**Conclusions:**

Serum DHEA and DHEAS were significantly and negatively associated with CHD in middle-aged and elderly men with T2DM. This study suggests potential roles of DHEA and DHEAS in CHD pathogenesis.

## Introduction

Cardiovascular disease (CVD) is currently the leading cause of death worldwide. Coronary heart disease (CHD) and stroke are two major forms of CVD, accounting for 84.9% of deaths combined (1). Evidence indicates that sex has different effects on the manifestations and prognosis of CVD (2–4). In men, CHD is the initial manifestation; however, in women, it can present as stroke or heart failure. It is widely accepted that male sex is a strong risk factor for CVD. Nevertheless, the sex-related gap narrows as the incidence of CHD rapidly increases, concurrent with the transition to menopause in women (5, 6). This sexual dimorphism indicates that sex hormones play an important role in the pathogenesis of CVD.

Dehydroepiandrosterone (DHEA) and the more abundant dehydroepiandrosterone sulfate (DHEAS) are important precursors of the active sex hormones testosterone and estradiol. DHEA has been hypothesized to reverse vascular remodeling, improve vascular endothelial cell function, and reduce oxidative stress (7–9). Previous studies have shown that, in men, low serum levels of DHEA increase the risk of CHD; however, the relationship between DHEAS and CHD is contradictory (10, 11). Among women, the associations of DHEA and DHEAS with CVD have also been found to be inconsistent (11–13). Additionally, diabetes has adverse effects on the reproductive system and sex hormones and increases the frequency of hypogonadism (14, 15). The risk of CVD among patients with diabetes is twice that of non-diabetic men and three times that of non-diabetic women (16). However, few studies have reported the associations of DHEA and DHEAS with CHD and stroke in patients with type 2 diabetes mellitus (T2DM).

Therefore, this cross-sectional study aimed to explore the associations of DHEA and DHEAS with CHD and stroke among middle-aged and elderly men and women with T2DM.

## Materials and Methods

### Study Subjects

**Figure 1:**
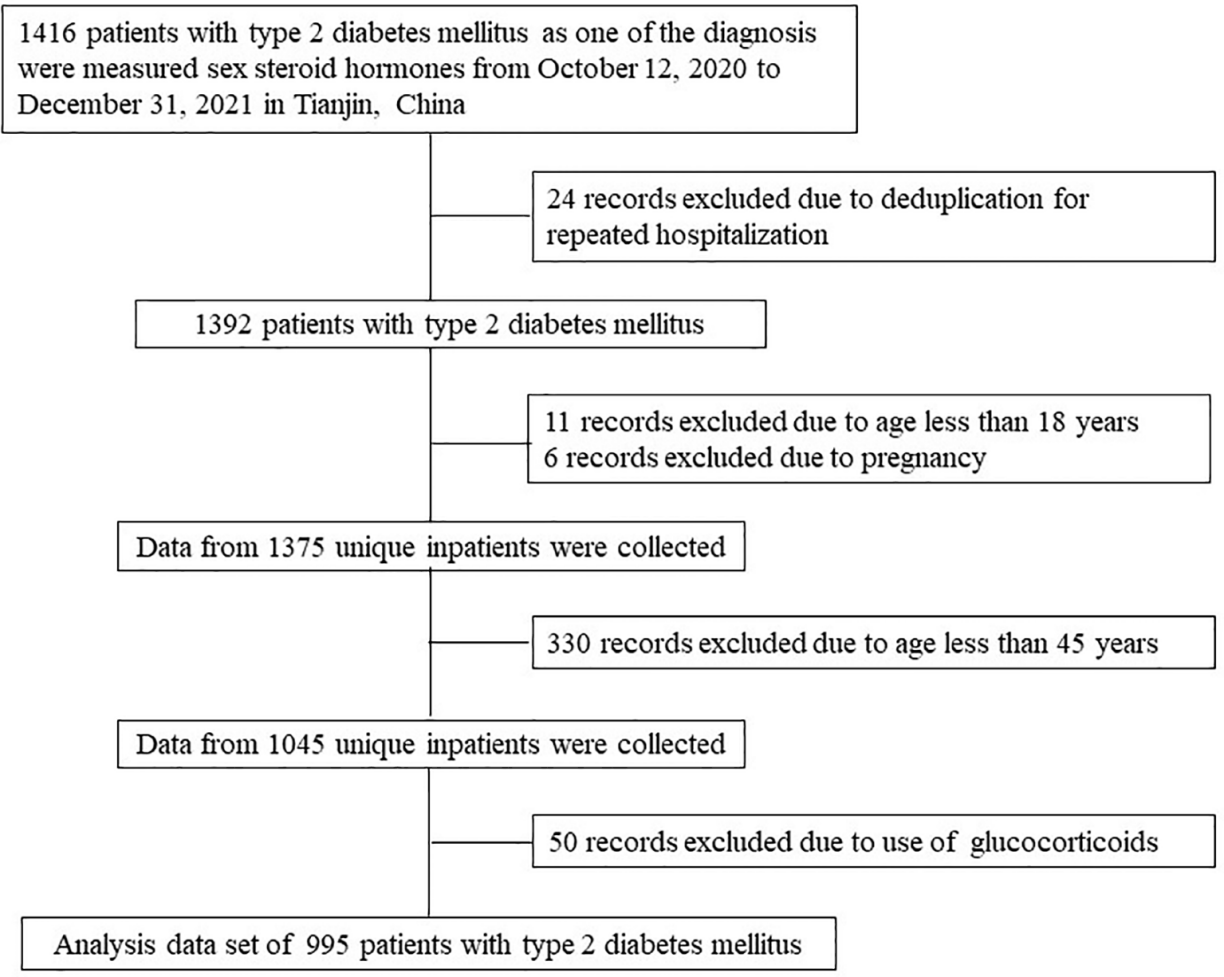
Flow chart of the identification of the study population. Based on the exclusion criteria, 995 patients with type 2 diabetes mellitus were included in the final analysis.

The medical records of 1,416 patients with T2DM who were hospitalized in the Department of Endocrinology and Metabolism, Tianjin Medical University General Hospital from October 12, 2020 to December 31, 2021 were reviewed, and then the serum levels of DHEA and DHEAS were measured in these subjects. If an individual had multiple hospitalization records, only one record was selected. Patients aged less than 45 years and pregnant women were excluded from the analysis. As the use of hormones is known to affect the levels of DHEA and DHEAS, it was also considered as a criterion for exclusion. **Figure 1** illustrates the population identification process. None of the participants enrolled in this study used sex hormones. In total, 995 patients with T2DM were included in the final analysis.

This study was approved by the Institutional Review Board of Tianjin Medical University General Hospital. The requirement for informed consent was waived (approval no. IRB2020-YX-027-01) because patient information was extracted from electronic medical records at the Department of Endocrinology and Metabolism; the identities of patients were also kept anonymous.

### Covariates

Data obtained from medical records included the following: age, sex, insurance type, cigarette smoking, alcohol consumption, medical history of diabetes, hypertension, height, weight, systolic blood pressure (SBP), diastolic blood pressure (DBP), fasting blood glucose (FBG), glycosylated hemoglobin (HbA1c), total cholesterol (TC), triglycerides (TG), high-density lipoprotein cholesterol (HDL-C), low-density lipoprotein cholesterol (LDL-C), and the use of glucagon-like peptide 1 (GLP-1) receptor agonists or sodium–glucose cotransporter 2 (SGLT-2) inhibitors.

Based on the type of medical insurance, participants were categorized into three groups: patients who had medical insurance for urban workers, or for non-working residents, or those who did not have any kind of medical insurance. The body mass index (BMI) was calculated as the participant’s weight divided by the square of the participant’s height (in kilograms per square meter).

### DHEA and DHEAS Measurements

Serum levels of DHEA and DHEAS were quantified using liquid chromatography–tandem mass spectrometry (LC-MS/MS) assays at the Laboratory of Endocrinology and Metabolism at Tianjin Medical University General Hospital. After admission, venous blood samples were collected in the morning after at least 10 h of fasting. Steroid extraction liquid (Hangzhou Calibra Diagnostics Co., Ltd., Zhejiang, China) was used to pretreat the samples. DHEA and DHEAS were measured using a Jasper™ HPLC system coupled to an AB SCIEX Triple Quad™ 4500MD mass spectrometer with a heated nebulizer ionization source in the positive ion mode. MultiQuant™ MD 3.0.2 software was used to quantify the data. The area ratios of the analyte to the internal standard peak of the six calibration standards were plotted to generate calibration curves for each set of samples; two quality control samples were included in each set of samples. The plot used linear regression with 1/*x* ^2^ weighting, and the correlation coefficients were greater than 0.99.

### Definitions

Diabetes was defined as a FBG level ≥7.0 mmol/L, a 2-h plasma glucose level ≥11.1 mmol/L, a HbA1c level ≥6.5%, a self-reported history of diabetes, or the use of hypoglycemic drugs (17). Hypertension was defined as SBP ≥140 mmHg, DBP ≥90 mmHg, self-reported history of hypertension, or the use of blood pressure-lowering drugs (18). CHD outcome was based on self-reported diagnoses, including myocardial infarction and angina or having a history of treatment for coronary artery bypass grafting or percutaneous coronary intervention. Stroke was based on self-reported diagnoses, including ischemic stroke and cerebral hemorrhage.

### Statistical Analyses

Continuous variables are shown as mean with standard deviation (SD) or median with interquartile range (IQR); categorical variables are presented as numbers with frequencies. Differences between the groups were evaluated using Student’s *t*-tests (continuous variables, normal distribution), Mann–Whitney *U* tests (continuous variables, skewed distribution), or chi-squared tests (categorical variables). Spearman’s correlations were used to determine the values and significance of the correlation coefficients between serum DHEA and DHEAS among men and women. Binary logistic regression analyses were conducted to assess the roles of DHEA and DHEAS in CHD and stroke, after adjusting for covariates in three models: model 1 was adjusted for age; model 2 was adjusted for model 1 plus current smoking, current drinking, and insurance type; and model 3 was adjusted for model 2 plus BMI, duration of T2DM, SBP, LDL-C, FBG, HbA1c, and the use of GLP-1 receptor agonists or SGLT-2 inhibitors. The levels of DHEAS and DHEA were also separately categorized into quartiles, and the lowest quartiles were used as the reference groups in the binary logistic regression. Restricted cubic splines were used to assess the dose–response associations of DHEA and DHEAS with CHD after adjusting for age, current smoking, current drinking, insurance type, BMI, duration of diabetes, SBP, LDL-C, FBG, HbA1c, and the use of GLP-1 receptor agonists or SGLT-2 inhibitors. The knots were placed at the 5th, 35th, 65th, and 95th percentiles, and the reference values were the 50th percentiles for DHEA and DHEAS based on the distribution. Receiver operating characteristic (ROC) curve analysis was performed to determine the cutoff values for DHEA and DHEAS for the detection of CHD in men with T2DM. The ROC curves are plots of sensitivity versus 1 − specificity for the DHEA and DHEAS levels of each patient. The Youden index (value of the maximum of sensitivity plus specificity − 1) of the ROC curve was used to confirm the optimal cutoff values for DHEA and DHEAS. The results are shown as odds ratios (ORs) and 95% confidence intervals (CIs). A two-tailed *p*-value <0.05 was considered as statistically significant. SPSS for Windows (version 25.0; Chicago, IL, USA) and R software (version 4.1.1; R Foundation) were used for the analyses.

## Results

### Clinical Characteristics of the Study Population

**Figure 2:**
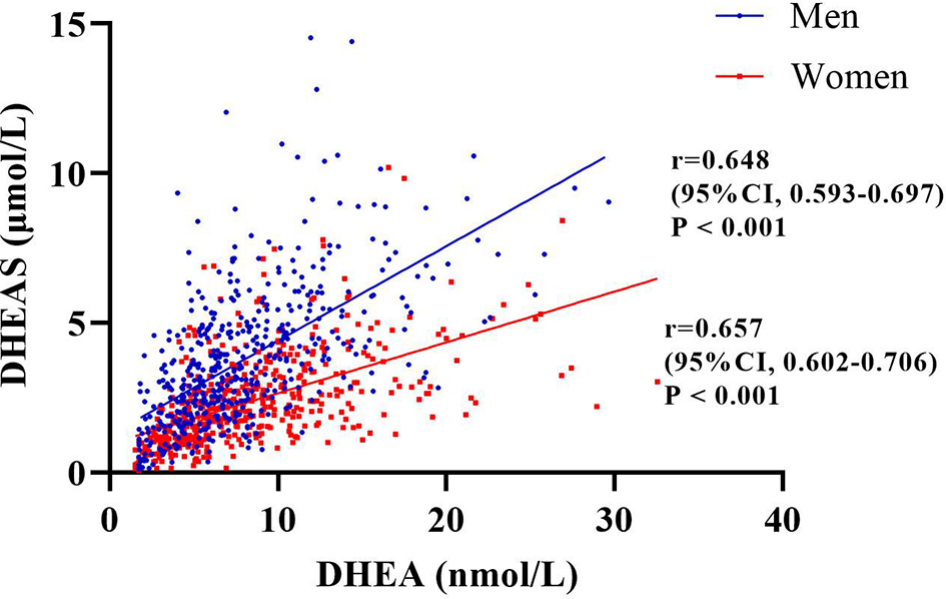
Correlation between serum dehydroepiandrosterone (DHEA) and dehydroepiandrosterone sulfate (DHEAS) among the men and women included in this study evaluated by Spearman’s correlations. The figure indicates that the serum levels of DHEA and DHEAS were highly collinear (among men: *r* = 0.648, 95% CI = 0.593–0.697, *p* < 0.001; among women: *r* = 0.657, 95% CI = 0.602–0.706, *p* < 0.001).

In this study, 51.3% of the patients were men (*n* = 510), and the mean age of the total population was 62.93 years. The prevalence rates of CHD and stroke were 16.1% and 18.5%, respectively. Most of the patients (83.3%) had medical insurance for urban workers. The median duration of T2DM was 10 years, and the mean HbA1c level of the total population was 8.39%. The median values of DHEA and DHEAS were 6.91 nmol/L and 2.56 μmol/L, respectively (**Table 1**). Furthermore, **Figure 2** indicates that the serum levels of DHEA and DHEAS were highly collinear (among men: *r* = 0.648, 95% CI = 0.593–0.697, *p* < 0.001; among women: *r* = 0.657, 95% CI = 0.602–0.706, *p* < 0.001).

**Table 1:** Characteristics of patients with type 2 diabetes mellitus.

	Men	Women	Total
Participants, *n* (%)	510 (51.3)	485 (48.7)	995 (100)
CHD, *n* (%)	106 (20.8)	54 (11.1)	160 (16.1)
Stroke, *n* (%)	94 (18.4)	90 (18.6)	184 (18.5)
Age (years)	62.01 ± 9.08	63.91 ± 8.84	62.93 ± 9.01
BMI (kg/m^2^)	26.08 ± 3.81	25.85 ± 4.31	25.97 ± 4.06
Current smoking, *n* (%)	225 (44.4)	22 (4.6)	247 (25.0)
Current drinking, *n* (%)	223 (43.9)	14 (2.9)	237 (24.0)
Insurance type, *n* (%)			
Urban workers	427 (84.1)	399 (82.4)	826 (83.3)
Non-working urban residents	50 (9.8)	63 (13.0)	113 (11.4)
Self-pay	31 (6.1)	22 (4.5)	53 (5.3)
Duration of type 2 diabetes (years)	10 (3–18)	10 (3–18)	10 (3–18)
Use of GLP-1 receptor agonists, *n* (%)	30 (6.1)	27 (5.7)	57 (5.9)
Use of SGLT-2 inhibitors, *n* (%)	56 (11.3)	34 (7.2)	90 (9.3)
Hypertension, *n* (%)	337 (66.1)	319 (65.8)	656 (65.9)
Blood pressure (mmHg)			
Systolic	137 ± 18	136 ± 19	136 ± 18
Diastolic	83 ± 11	79 ± 10	81 ± 11
TC (mmol/L)	4.65 ± 1.30	5.05 ± 1.46	4.84 ± 1.39
TG (mmol/L)	1.60 (1.13–2.30)	1.65 (1.21–2.17)	1.63 (1.18–2.26)
HDL-C (mmol/L)	1.06 ± 0.28	1.15 ± 0.29	1.10 ± 0.29
LDL-C (mmol/L)	2.80 ± 0.87	2.97 ± 1.03	2.88 ± 0.96
FBG (mmol/L)	7.45 ± 2.91	7.43 ± 2.67	7.44 ± 2.80
HbA1c (%)	8.51 ± 2.14	8.25 ± 1.97	8.39 ± 2.06
DHEA (nmol/L)	6.72 (4.69–10.06)	6.99 (4.59–10.84)	6.91 (4.62–10.37)
DHEAS (μmol/L)	3.26 (2.00–4.90)	2.02 (1.21–3.03)	2.56 (1.54–4.15)

CHD, coronary heart disease; BMI, body mass index; GLP-1, glucagon-like peptide 1; SGLT-2, sodium–glucose cotransporter 2; TC, total cholesterol; TG, triglycerides; HDL-C, high-density lipoprotein cholesterol; LDL-C, low-density lipoprotein cholesterol; FBG, fasting blood glucose; HbA1c, glycosylated hemoglobin; DHEA, dehydroepiandrosterone; DHEAS, dehydroepiandrosterone sulfate.

### Prevalence of CHD and Stroke by Quartiles of DHEA and DHEAS

**Figure 3:**
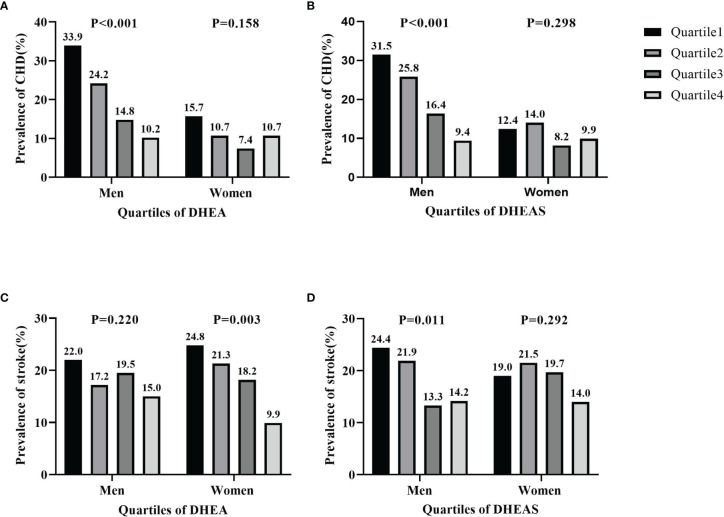
Prevalence of coronary heart disease (CHD) and stroke by quartiles of serum dehydroepiandrosterone (DHEA) and dehydroepiandrosterone sulfate (DHEAS) in men and women. **(A, B)** Prevalence of CHD by DHEA **(A)** and by DHEAS (**B**). **(C, D)** Prevalence of stroke by DHEA **(C)** and by DHEAS (**D**). The figure shows that the percentages of men with CHD significantly decreased in accordance with increasing quartiles of serum DHEA and DHEAS (all *p* < 0.001). The prevalence of stroke significantly decreased in line with increasing quartiles of serum DHEA in women (*p* = 0.003) and DHEAS in men (*p* = 0.011).

The prevalence of CHD and stroke according to quartiles of serum DHEA and DHEAS in men and women is shown in **Figure 3**. The percentage of men with CHD significantly decreased with increasing quartiles of serum DHEA (33.9% in quartile 1, 24.2% in quartile 2, 14.8% in quartile 3, and 10.2% in quartile 4; *p* < 0.001) and DHEAS (31.5% in quartile 1, 25.8% in quartile 2, 16.4% in quartile 3, and 9.4% in quartile 4; *p* < 0.001). Moreover, the prevalence of stroke significantly decreased with increasing quartiles of serum DHEA in women (24.8% in quartile 1, 21.3% in quartile 2, 18.2% in quartile 3, and 9.9% in quartile 4; *p* = 0.003) and DHEAS in men (24.4% in quartile 1, 21.9% in quartile 2, 13.3% in quartile 3, and 14.2% in quartile 4; *p* = 0.011).

### Associations of DHEA and DHEAS With the Risk of CHD and Stroke

**Table 2** shows the ORs of CHD by DHEA and DHEAS status in a binary logistic regression analysis in patients with T2DM. In men with T2DM, after adjusting for potential confounders in model 3, the risk of CHD decreased with increasing levels of serum DHEA (OR = 0.38, quartile 4 *vs*. quartile 1; 95% CI = 0.16–0.90; *p* = 0.037 for trend). Consistently, when taken as a continuous variable, per SD increment of serum DHEA was significantly related to a 41% decrease in the risk of CHD among men in the fully adjusted model (OR = 0.59, 95% CI = 0.40–0.87, *p* < 0.05). The serum level of DHEAS was also inversely related to the risk of CHD among men when taken as a continuous variable in model 3 (OR = 0.56, 95% CI = 0.38–0.82, *p* < 0.05). Similarly, this relationship remained statistically significant when DHEAS was categorized into quartiles (OR = 0.27, quartile 4 *vs*. quartile 1; 95% CI = 0.11–0.67; *p* = 0.018 for trend). In women with T2DM, no significant relationships were observed between increasing levels of DHEA and DHEAS and risk of CHD (all *p* > 0.05).

**Table 2:** Odds ratios of CHD by different DHEA and DHEAS status in patients with type 2 diabetes mellitus.

	Odds ratios (95% CI)		
	Model 1	Model 2	Model 3
Men			
DHEA (nmol/L)			
Quartile 1	Reference	Reference	Reference
Quartile 2	0.68 (0.39–1.19)	0.68 (0.39–1.19)	0.93 (0.46–1.88)
Quartile 3	**0.39 (0.21–0.73)**	**0.38 (0.20–0.71)**	**0.44 (0.20–0.96)**
Quartile 4	**0.28 (0.14–0.58)**	**0.27 (0.13–0.57)**	**0.38 (0.16–0.90)**
*p* for trend	**0.001**	**0.001**	**0.037**
Per SD increment	**0.54 (0.39–0.74)**	**0.53 (0.38–0.74)**	**0.59 (0.40–0.87)**
DHEAS (μmol/L)			
Quartile 1	Reference	Reference	Reference
Quartile 2	0.84 (0.48–1.46)	0.85 (0.48–1.49)	0.63 (0.31–1.26)
Quartile 3	**0.50 (0.27–0.93)**	**0.50 (0.27–0.92)**	**0.38 (0.18–0.83)**
Quartile 4	**0.29 (0.14–0.60)**	**0.28 (0.13–0.58)**	**0.27 (0.11–0.67)**
*p* for trend	**0.003**	**0.003**	**0.018**
Per SD increment	**0.62 (0.47–0.83)**	**0.61 (0.46–0.81)**	**0.56 (0.38–0.82)**
Women			
DHEA (nmol/L)			
Quartile 1	Reference	Reference	Reference
Quartile 2	0.82 (0.37–1.79)	0.86 (0.39–1.92)	1.37 (0.50–3.78)
Quartile 3	0.58 (0.25–1.38)	0.61 (0.25–1.49)	0.53 (0.15–1.84)
Quartile 4	0.91 (0.41–2.01)	1.00 (0.45–2.26)	1.65 (0.60–4.49)
*p* for trend	0.659	0.700	0.343
Per SD increment	1.06 (0.80–1.40)	1.08 (0.81–1.44)	1.06 (0.73–1.53)
DHEAS (μmol/L)			
Quartile 1	Reference	Reference	Reference
Quartile 2	1.36 (0.63–2.94)	1.45 (0.66–3.18)	1.97 (0.69–5.65)
Quartile 3	0.93 (0.39–2.25)	0.84 (0.34–2.10)	1.50 (0.48–4.68)
Quartile 4	1.08 (0.47–2.50)	1.11 (0.47–2.60)	1.59 (0.52–4.82)
*p* for trend	0.804	0.638	0.653
Per SD increment	0.95 (0.70–1.28)	0.95 (0.70–1.29)	1.08 (0.76–1.53)

Model 1: adjusted for age; model 2: model 1 + current smoking, current drinking, and insurance type; model 3: model 2 + BMI, duration of diabetes, SBP, LDL-C, FBG, HbA1c, and the use of GLP-1 receptor agonists or SGLT-2 inhibitors

CI, confidence interval; CHD, coronary heart disease; DHEA, dehydroepiandrosterone; DHEAS, dehydroepiandrosterone sulfate; SD, standard deviation; BMI, body mass index; SBP, systolic blood pressure; LDL-C, low-density lipoprotein cholesterol; FBG, fasting blood glucose; HbA1c, glycosylated hemoglobin, GLP-1, glucagon-like peptide 1; SGLT-2, sodium–glucose cotransporter 2.

Bold results are statistically significant.

The associations of DHEA and DHEAS with stroke are shown in **Table 3**. When considered as a continuous variable, per SD increment of serum DHEA was significantly related to a 27% decrease in the risk of stroke among women in model 1 (OR = 0.73, 95% CI = 0.55–0.96, *p* < 0.05), but this association was not statistically significant in the fully adjusted model (OR = 0.83, 95% CI = 0.62–1.12, *p* > 0.05). No significant relationships were found between DHEA, DHEAS, and stroke in male patients (all *p* > 0.05).

**Table 3:** Odds ratios of stroke by different DHEA and DHEAS status in patients with type 2 diabetes mellitus.

	Odds ratios (95% CI)		
	Model 1	Model 2	Model 3
**Men**			
DHEA (nmol/L)			
Quartile 1	Reference	Reference	Reference
Quartile 2	0.86 (0.45–1.62)	0.87 (0.45–1.67)	0.81 (0.38–1.74)
Quartile 3	1.11 (0.59–2.08)	1.12 (0.59–2.12)	1.10 (0.52–2.32)
Quartile 4	0.98 (0.49–1.96)	1.00 (0.49–2.04)	0.98 (0.43–1.73)
* p* for trend	0.981	0.905	0.893
Per SD increment	0.97 (0.74–1.26)	0.98 (0.75–1.28)	0.91 (0.66–1.27)
DHEAS (μmol/L)			
Quartile 1	Reference	Reference	Reference
Quartile 2	1.01 (0.55–1.83)	0.98 (0.54–1.81)	0.92 (0.44–1.92)
Quartile 3	0.59 (0.30–1.16)	0.57 (0.29–1.13)	0.45 (0.20–1.00)
Quartile 4	0.72 (0.36–1.42)	0.67 (0.34–1.34)	0.66 (0.28–1.53)
* p* for trend	0.340	0.297	0.201
Per SD increment	0.91 (0.70–1.18)	0.90 (0.69–1.17)	0.83 (0.60–1.14)
**Women**			
DHEA (nmol/L)			
Quartile 1	Reference	Reference	Reference
Quartile 2	0.98 (0.53–1.81)	1.04 (0.55–1.95)	1.14 (0.54–2.43)
Quartile 3	0.84 (0.44–1.59)	0.88 (0.45–1.71)	1.18 (0.53–2.61)
Quartile 4	**0.42 (0.20–0.88**)	**0.45 (0.21–0.95)**	0.63 (0.27–1.44)
* p* for trend	0.101	0.132	0.436
Per SD increment	**0.73 (0.55**–**0.96)**	**0.74 (0.57–0.98)**	0.83 (0.62–1.12)
DHEAS (μmol/L)			
Quartile1	Reference	Reference	Reference
Quartile2	1.31 (0.69–2.50)	1.50 (0.77–2.90)	1.62 (0.72–3.64)
Quartile3	1.41 (0.72–2.75)	1.55 (0.78–3.07)	1.89 (0.84–4.25)
Quartile4	0.88 (0.44–1.79)	0.93 (0.45–1.93)	1.11 (0.47–2.63)
* p* for trend	0.491	0.332	0.350
Per SD increment	0.96 (0.75–1.22)	0.97 (0.76–1.24)	1.01 (0.76–1.34)

Model 1: adjusted for age; model 2: model 1 + current smoking, current drinking and insurance type; model 3: model 2 + BMI, duration of diabetes, SBP, LDL-C, FBG, HbA1c, and the use of GLP-1 receptor agonists or SGLT-2 inhibitors

CI, confidence interval; DHEA, dehydroepiandrosterone; DHEAS, dehydroepiandrosterone sulfate; SD, standard deviation; BMI, body mass index; SBP, systolic blood pressure; LDL-C, low-density lipoprotein cholesterol; FBG, fasting blood glucose; HbA1c, glycosylated hemoglobin; GLP-1, glucagon-like peptide 1; SGLT-2, sodium–glucose cotransporter 2.

Bold results are statistically significant.

**Figure 4:**
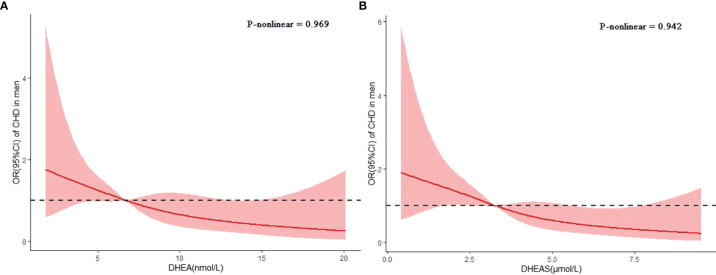
Associations of dehydroepiandrosterone (DHEA) and dehydroepiandrosterone sulfate (DHEAS) with coronary heart disease (CHD) in men with type 2 diabetes mellitus. Restricted cubic splines were used to assess the dose–response associations of DHEA **(A)** and DHEAS **(B)** with CHD after adjusting for age, current smoking, current drinking, insurance type, body mass index (BMI), duration of diabetes, systolic blood pressure (SBP), low-density lipoprotein cholesterol (LDL-C), fasting blood glucose (FBG), glycosylated hemoglobin (HbA1c), and the use of glucagon-like peptide 1 (GLP-1) receptor agonists or sodium–glucose cotransporter 2 (SGLT-2) inhibitors. The *p*-values for nonlinear associations were 0.969 and 0.942 for DHEA and DHEAS, respectively.

In the spline regression analyses, the risk of CHD decreased progressively with the serum levels of DHEA and DHEAS after adjusting for age, current smoking, current drinking, insurance type, BMI, duration of diabetes, SBP, LDL-C, FBG, HbA1c, and use of GLP-1 receptor agonists or SGLT-2 inhibitors (**Figure 4**). The *p*-values for nonlinear associations were 0.969 for DHEA and 0.942 for DHEAS, indicating that the associations of DHEA and DHEAS with CHD were linear.

**Figure 5:**
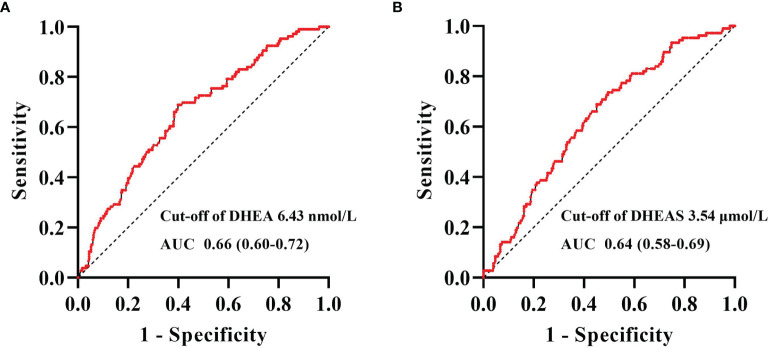
Receiver operating characteristic (ROC) curve analysis of dehydroepiandrosterone (DHEA) and dehydroepiandrosterone sulfate (DHEAS) to recognize coronary heart disease (CHD) in men with type 2 diabetes mellitus. ROC curve analysis revealed that the areas under the curve (AUCs) for DHEA **(A)** and DHEAS **(B)** were 0.66 (95% CI = 0.60–0.72) and 0.64 (95% CI = 0.58–0.69), respectively. The optimal cutoff values, with the best trade-off between sensitivity and specificity, were 6.43 nmol/L for DHEA and 3.54 μmol/L for DHEAS.

**Figure 5** shows the ROC curve analyses of the levels of DHEA and DHEAS for identifying CHD in men with T2DM. The areas under the curve (AUCs) of DHEA and DHEAS were 0.66 (95% CI = 0.60–0.72) and 0.64 (95% CI = 0.58–0.69), respectively. The optimal cutoff values allowing for the best trade-off between sensitivity and specificity were 6.43 nmol/L for DHEA and 3.54 μmol/L for DHEAS.

## Discussion

In this study, we found that the serum levels of DHEA and DHEAS were significantly and negatively associated with CHD in middle-aged and elderly men with T2DM after adjusting for potential confounding factors. The optimal cutoff values to detect CHD in men with T2DM were 6.43 nmol/L for DHEA and 3.54 μmol/L for DHEAS. In contrast, no significant associations were found between DHEA and DHEAS on the one hand and stroke on the other in middle-aged and elderly men and women with T2DM.

DHEA, an important precursor of testosterone and estradiol, is mainly synthesized by the adrenal cortex and gonads. Previous studies have shown that lower serum levels of DHEA are associated with a greater risk of CHD in men. A prospective study recruiting men aged 40–70 years reported that lower serum levels of DHEA were related to the incidence of ischemic heart disease, independent of traditional risk factors (19). In the Osteoporotic Fractures in Men Study, serum DHEA was found to predict the risk of developing CHD among elderly men (10). Moreover, a cross-sectional study involving patients with T2DM from China showed that serum levels of DHEA were inversely related to the odds of CVD in male patients, defined by CHD, myocardial infarction, or stroke (20). However, the association of DHEA with CHD in women was inconsistent. A prospective study revealed that postmenopausal women with low levels of serum DHEA were more likely to incur myocardial infarction after adjustment for potential confounding factors (13). In contrast, the Rotterdam Study showed no statistically significant association between the levels of DHEA and the incidence of CVD, including CHD and stroke (12). In patients with T2DM from China, DHEA was not significantly associated with CVD risk (20). Consistently, in this hospital-based cross-sectional study, we found that low serum levels of DHEA were associated with CHD in middle-aged and elderly men, but not in women with T2DM. In addition, the optimal cutoff value for DHEA to detect CHD in men with T2DM was determined to be 6.43 nmol/L.

It has been shown that DHEA regulates vascular remodeling and endothelial function through different mechanisms. Endothelial cells (ECs) and smooth muscle cells (SMCs) are important cells of the blood vessel in the progression of vascular disease. DHEA has been shown to upregulate nitric oxide (NO) synthase and improve the bioavailability of NO in ECs, resulting in elevated cyclic guanosine monophosphate production and subsequent relaxation in SMCs (8, 21). DHEA has also been shown to reverse systemic vascular remodeling in human carotid vascular SMCs (7). In the cardiovascular system, DHEA acts as an antioxidant and protects cultured human ECs from injury induced by superoxide (22). Notably, evidence on DHEA supplementation has yielded controversial results. DHEA treatment can reduce myocardial hypertrophy by upregulating the expression of the sigma-1 receptor in ovariectomized rats (23). Randomized, double-blind trials with small sample sizes showed that 25 or 50 mg of DHEA replacement per day improved vascular endothelial function, indices of arterial stiffness, lipid profile, and insulin sensitivity (24–26). However, other studies have shown that DHEA replacement has no effect on the cardiovascular parameters or endothelial function in patients with adrenal insufficiency (27, 28). Taken together, clinical trials with larger sample sizes are needed to clarify the significance of DHEA supplementation in the prevention of CHD.

DHEAS, the sulfated form of DHEA, is the most abundant steroid hormone in human circulation. In the present study, we found that serum DHEAS was negatively associated with CHD in men with T2DM after adjustment for potential confounding factors and that the optimal cutoff value for DHEAS to detect CHD in men with T2DM was 3.54 μmol/L. Previous epidemiological studies on the relationship between DHEAS and CHD have shown contradictory results. Low serum levels of DHEAS at baseline were found to predict the development of ischemic heart disease in middle-aged men in the Massachusetts Male Aging Study (19). A prospective cohort study in Sweden showed that a low level of DHEAS was associated with ischemic heart disease mortality in elderly men (29). Moreover, among women, lower serum levels of DHEAS were found to be independently associated with the risk of death from CVD, including CHD, stroke, heart failure, or peripheral vascular diseases (30). Nevertheless, a community-based study in Framingham reported that, in men, baseline levels of DHEAS were not statistically related to the risk of developing CVD (31). In a community-based study recruiting 8,143 participants, no statistically significant association was found between low levels of DHEAS and the risk of CHD in men or women (11). The differences in the study design, the included population, outcome measures, and assays of the hormones detected could partially explain the inconsistencies in the results of these studies.

In this cross-sectional study, the serum levels of DHEA and DHEAS were not statistically related to the risk of stroke, including ischemic stroke and cerebral hemorrhage, in middle-aged and elderly men and women with T2DM. Consistent with our study, in the prospective study mentioned above, serum DHEA and DHEAS were independent predictors of CHD, but not stroke, in elderly men (10). Furthermore, in young women with polycystic ovary syndrome, DHEAS was not significantly associated with carotid intima–media thickness, which is considered to predict the incidence of myocardial infarction and stroke (32). However, in the Nurses’ Health Study, low serum DHEAS was confirmed to be associated with a high risk of ischemic stroke among women, following a nested case–control design (33). In postmenopausal women who suffer an acute stroke, low DHEAS was found to be significantly associated with disease severity (34). In a prospective study involving patients with acute ischemic stroke, DHEAS, but not DHEA, predicted the 1-year functional outcomes in men and women (35). The differences in the outcomes measured, as well as the specific participants included, could be partly responsible for the conflicting results. Therefore, further prospective research in the general population is needed to better clarify the effects of DHEA and DHEAS on stroke.

Our study had several limitations. Firstly, the sample size of 510 men and 485 women was relatively small. However, some epidemiological studies have also been conducted with relatively small sample sizes (36, 37), which also evaluated the concentration of sex hormones *via* LC-MS/MS. Secondly, owing to the cross-sectional nature of this study, we could not identify causality. Thirdly, some confounding factors, such as physical activity and dietary habits, were not included in our analysis because the data were extracted from medical records. Finally, we examined the association of DHEA and DHEAS with CHD and stroke in middle-aged and elderly participants with T2DM; therefore, our findings cannot be generalized to other populations.

In conclusion, we found that the serum levels of DHEA and DHEAS were significantly and negatively associated with CHD in middle-aged and elderly men with T2DM. In contrast, no significant associations were observed between DHEA and DHEAS on the one hand and stroke on the other in patients with T2DM. The results from this study suggest potential roles of DHEA and DHEAS in the pathogenesis of CHD.

## Data Availability Statement

The raw data supporting the conclusions of this article will be made available by the authors, without undue reservation.

## Ethics Statement

The studies involving human participants were reviewed and approved by the institutional review board of Tianjin Medical University General Hospital. Written informed consent from the patients/participants’ legal guardian/next of kin was not required to participate in this study in accordance with the national legislation and the institutional requirements.

## Author Contributions

XZ, JX, and TL designed the research, collected clinical data, and wrote the manuscript. QH and JC were involved in clinical data collection. ST, XL, and ML designed the study and revised the paper. All authors contributed to the article and approved the submitted version.

## Funding

This work was supported by the National Natural Science Foundation of China (81830025 and 81620108004) and the National Key R&D Program of China (2019YFA0802502).

## Conflict of Interest

The authors declare that the research was conducted in the absence of any commercial or financial relationships that could be construed as a potential conflict of interest.

## Publisher’s Note

All claims expressed in this article are solely those of the authors and do not necessarily represent those of their affiliated organizations, or those of the publisher, the editors and the reviewers. Any product that may be evaluated in this article, or claim that may be made by its manufacturer, is not guaranteed or endorsed by the publisher.
